# Supplementary material for: Inferring microbial interactions with their environment from genomic and metagenomic data
Source: PLoS Comput Biol. 2023 Nov 13;19(11):e1011661. doi: 10.1371/journal.pcbi.1011661 (PMC10681327; doi:10.1371/journal.pcbi.1011661)
Supplement: S1 Text — (PDF) [file pcbi.1011661.s002.pdf]

Surfin FBA Technical Details.

James D. Brunner[1,2], Laverne A. Gallegos-Graves[1], and Marie E. Kroeger[1]

[1]**Biosciences Division, Los Alamos National Laboratory**
[2]**Center for Nonlinear Studies, Los Alamos National Laboratory**

In this supplemental document, we set out the technical details that underlie the "Surfin FBA" method. We present these details in the context of a linear program in so-called "standard form" (eq. (1)), rather than the more familiar form that appears in genome-scale modeling literature, or the form that appears in the main text of the manuscript. However, any linear program can be easily reformulated in standard form using simple algebraic tricks, including separating positive and negative values (which, in the case of FBA, simply means treating forward and reverse reactions as separate) so that all variables are constrained to be non-negative, and introducing "slack" variables so that constraints take the form of equality rather than inequality. For details on linear programming, see, for example, [1].

1 NOTATION

Let $\boldsymbol{c} \in \mathbb{R}^m$, $\boldsymbol{b} \in \mathbb{R}^n$, and $A \in \mathbb{R}^{n \times m}$.

Consider the primal linear program in standard form

$$\begin{array}{l} \min \boldsymbol{x} \cdot \boldsymbol{c} \\ A\boldsymbol{x} = \boldsymbol{b} \\ \boldsymbol{x} \geq 0 \end{array} \tag{1}$$

and the basic set β for a basic optimal solution $\overline{\boldsymbol{x}}$ defined as

$$\overline{x}_i = \begin{cases} (\boldsymbol{x}_\beta)_{j:\beta_j = i} & i \in \beta \\ 0 & i \notin \beta \end{cases} \tag{2}$$

where $\boldsymbol{x}_\beta = A_\beta^{-1}\boldsymbol{b}$ and A_β is the matrix formed by the columns of A corresponding to the index set β.

Furthermore, note that if $\boldsymbol{x}_\beta > \boldsymbol{0}$, then β is simply the index set of the non-zero entries in the basic optimal solution $\overline{\boldsymbol{x}}$. However, if there are fewer than n non-zero entries in the basic optimal solution $\overline{\boldsymbol{x}}$ than a choice of basic index set β is ambiguous. In fact, *any* combination of indices which contains the set of indices of the non-zero entries of $\overline{\boldsymbol{x}}$ may be used, provided the corresponding columns of A form a basis for $\mathbb{R}^n$.

Note that $\overline{\boldsymbol{x}}'$ as defined with $\boldsymbol{x}'_\beta = A_\beta^{-1}\boldsymbol{b}'$ is a solution to the linear program

$$\begin{array}{l} \min \boldsymbol{x} \cdot \boldsymbol{c} \\ A\boldsymbol{x} = \boldsymbol{b}' \\ \boldsymbol{x} \geq 0 \end{array} \tag{3}$$

as long as $\boldsymbol{b}' \in \text{cone}(A_\beta)$, meaning $\boldsymbol{x}'_\beta \geq \boldsymbol{0}$.

Suppose that the basic optimal solution $\overline{\boldsymbol{x}}$ has exactly $l \leq n$ non-zero entries. Let $\hat{\beta}$ be the indices of these entries. In order to form a basic index set, we may choose any $n-l$ indices $\tilde{\beta}$ such that the columns of A corresponding to $\beta = \hat{\beta} \cup \tilde{\beta}$ forms a basis for $\mathbb{R}^n$. Let $A_{\hat{\beta}}$ and $A_{\tilde{\beta}}$ denote the matrices formed by the columns of A corresponding to the indices $\hat{\beta}$ and $\tilde{\beta}$, respectively. Let $S_{\hat{\beta}}$ and $S_{\tilde{\beta}}$ denote the column space of $A_{\hat{\beta}}$ and $A_{\tilde{\beta}}$, respectively. Likewise, let C_β, $C_{\hat{\beta}}$ and $C_{\tilde{\beta}}$ denote the cone formed by the columns of A_β, $A_{\hat{\beta}}$ and $A_{\tilde{\beta}}$, respectively.

2 PROBLEM

Consider the dynamic linear program

$$\begin{array}{l} \min \boldsymbol{x}(t) \cdot \boldsymbol{c} \\ A\boldsymbol{x}(t) = \boldsymbol{b}(t) \\ \boldsymbol{x}(t) \geq 0 \end{array} \tag{4}$$

and assume that at $t=0$, the basic optimal solution $\overline{\boldsymbol{x}}(0)$ has exactly $l \leq n$ non-zero entries. We wish to choose a basic index set $\beta = \hat{\beta} \cup \tilde{\beta}$ so that there exists $t_1 > 0$ where $\boldsymbol{x}_\beta(t) = A_\beta^{-1}\boldsymbol{b}(t)$ corresponds to a basic optimal solution $\overline{\boldsymbol{x}}(t)$ to eq. (4) for $t \in [0, t_1]$, if such a basic index set exists.

3 CHOOSING β

3.1 Only one possibility: $l = n$

First, we note that if $l = n$, there is only one choice of basic index set. Furthermore,

$$\boldsymbol{b}(0) = A_\beta \boldsymbol{x}_\beta(0)$$

and using a power series expansion,

$$\boldsymbol{x}_\beta(t) = A_\beta^{-1}(\boldsymbol{b}(0) + \dot{\boldsymbol{b}}(0)\Delta t + o(\Delta t))$$

so

$$\boldsymbol{x}_\beta(t) = A_\beta^{-1}(\boldsymbol{b}(0)) + \Delta t A_\beta^{-1}(\dot{\boldsymbol{b}}(0)) + o(\Delta t)) = \boldsymbol{x}_\beta(0) + \Delta t A_\beta^{-1}(\dot{\boldsymbol{b}}(0)) + o(\Delta t)).$$

Because $\boldsymbol{x}_\beta(0) > \mathbf{0}$, we can conclude that there exists t_1 small enough so that for $t \in [0, t_1]$, we have $\boldsymbol{x}_\beta(t) \geq \mathbf{0}$ as long as $\dot{\boldsymbol{b}}(0)$ is finite. Therefore, if $l = n$ and there are some reasonable bounds on the smoothness of $\boldsymbol{b}(t)$ (i.e. Lipschitz continuity on an interval around $t = 0$).

3.2 More than one possibility: $l < n$

Next, we consider the case in which $l < n$, and we must choose a set $\tilde{\beta}$ to complete a basic index set β. Once again, we may write

$$\boldsymbol{x}_\beta(t) = \boldsymbol{x}_\beta(0) + \Delta t A_\beta^{-1}(\dot{\boldsymbol{b}}(0)) + o(\Delta t)) \tag{5}$$

and so see that the desired interval exists if

$$\left(A_\beta^{-1}\dot{\boldsymbol{b}}(0)\right)_j \geq 0 \tag{6}$$

for $j \in \tilde{\beta}$ (and again we have for example Lipschitz continuity of $\boldsymbol{b}(t)$). Writing

$$\boldsymbol{\omega}_\beta = A_\beta^{-1}\dot{\boldsymbol{b}}(0)$$

and

$$\dot{\boldsymbol{b}}(0) = A_{\hat{\beta}}\boldsymbol{\omega}_{\hat{\beta}} + A_{\tilde{\beta}}\boldsymbol{\omega}_{\tilde{\beta}}$$

we see that we must choose $\tilde{\beta}$ so that

$$\dot{\boldsymbol{b}}(0) \in S_{\hat{\beta}} \times C_{\tilde{\beta}}. \tag{7}$$

Let $P_{S_{\hat{\beta}}^\perp}$ be the orthogonal projection operator onto $S_{\hat{\beta}}^\perp$, the space orthogonal to $S_{\hat{\beta}}$. Then

$$P_{S_{\hat{\beta}}^\perp}\dot{\boldsymbol{b}}(0) = P_{S_{\hat{\beta}}^\perp}A_{\tilde{\beta}}\omega_{\tilde{\beta}}.$$

Note that because A_β forms a basis for $\mathbb{R}^n$, the columns of $P_{S_{\hat{\beta}}^\perp}A_{\tilde{\beta}}$ form a basis for $S_{\hat{\beta}}^\perp$. We can conclude that $\dot{\boldsymbol{b}}(0)$ satisfies eq. (7) if and only if $P_{S_{\hat{\beta}}^\perp}\dot{\boldsymbol{b}}(0)$ is in the cone formed by the columns of $P_{S_{\hat{\beta}}^\perp}A_{\tilde{\beta}}$.

To find a set $\tilde{\beta}$ such that $\tilde{\boldsymbol{b}} = P_{S_{\hat{\beta}}^\perp}\dot{\boldsymbol{b}}(0)$ is in the cone formed by the columns of $P_{S_{\hat{\beta}}^\perp}A_{\tilde{\beta}}$, we can project each column of A that is not in $A_{\hat{\beta}}$ onto $S_{\hat{\beta}}^\perp$ and call the resulting matrix $\tilde{A}$. Then we seek a vector $\tilde{\boldsymbol{\omega}}$ such that $\tilde{A}\tilde{\boldsymbol{\omega}} = \tilde{\boldsymbol{b}}$, $\tilde{\boldsymbol{\omega}} \geq 0$, and $\tilde{\boldsymbol{\omega}}$ has at most $n - l$ non-zero entries. To find such a vector, we may simply find a basic feasible solution to any LP with the constraints

$$\begin{aligned} \tilde{A}\tilde{\boldsymbol{\omega}} &= \tilde{\boldsymbol{b}} \\ \tilde{\boldsymbol{\omega}} &\geq 0. \end{aligned} \tag{8}$$

4 EFFICIENT IMPLEMENTATION

Projection of large matrices onto subspaces with non-orthogonal bases is very unstable and generally a bad idea. To avoid this, we can use a custom simplex algorithm on the so-called "phase-one" problem that finds a basic feasible solution $\boldsymbol{\omega}$ such that $\tilde{\boldsymbol{\omega}} = P_{S_{\hat{\beta}}^\perp}\boldsymbol{\omega}$ satisfies eq. (8).

Precisely, we seek a basic feasible solution (and associated basis) to the problem

$$\begin{aligned} A\boldsymbol{\omega} &= \dot{\boldsymbol{b}} \\ \boldsymbol{\omega}_j &\geq 0 \quad j \notin \hat{\beta} \end{aligned} \tag{9}$$

such that $\hat{\beta}$ is a subset of the associated basic index set. We can find a basic feasible solution to eq. (9) by solving the phase-one problem:

$$\begin{aligned} &\min \omega_{n+1} \\ &A\boldsymbol{\omega} + A_{n+1}\omega_{n+1} = \dot{\boldsymbol{b}} \\ &\omega_j, \omega_{n+1} \geq \mathbf{0} \qquad\qquad j \notin \hat{\beta} \end{aligned} \tag{10}$$

where

$$A_{n+1} = -A_{\beta}\mathbf{1}. \tag{11}$$

To solve eq. (10), we first find a basic feasible solution by pivoting $n+1$ into the basic index set, swapping it for $i^* \notin \hat{\beta}$ such that $\overline{\omega}_{i^*}$ is most negative. Then we simply perform the standard simplex algorithm with the modification that we do not pivot out any member of $\hat{\beta}$. This terminates when we are able to pivot out $n+1$.

4.1 Minimizing re-optimizations

In order to minimize the number of re-optimizations we need to compute, we can attempt to make as many of the $\omega_j > 0$ as possible, including $j \in \hat{\beta}$, and at least make ω_j, $j \in \hat{\beta}$, not too negative. We may even think about the magnitude of $x_j > 0$ to prioritize which ω_j shouldn't be too negative.

We can estimate how soon a re-optimization will be necessary using the x_j and ω_j by noting that for $x_j > 0$, a linear estimate of the time l_j until $x_j = 0$ is

$$l_j = -\frac{x_j}{\omega_j}$$

if $\omega_j < 0$, and otherwise $l_j = \infty$. Clearly, we want to maximize the smallest such l_j. To do this without treating $\omega_j > 0$ and $\omega_j < 0$ differently, we can instead take

$$s_j(\boldsymbol{\omega}) = \frac{1}{l_j} = -\frac{\omega_j}{x_j}$$

where $x_j \neq 0$, and otherwise $s_j = 0$. Here, we write s_j as a function of $\boldsymbol{\omega}$ to emphasize that we $\boldsymbol{x}$ is determined by the initial optimization and cannot be changed, while determining $\boldsymbol{\omega}$ determines the basis used to define $\boldsymbol{x}$. Then, we can increase the time to the next optimization by minimizing the largest s_j over the feasible choices of $\boldsymbol{\omega}$. The problem is then

$$\begin{gathered} \min_{\boldsymbol{\omega}} \left(\max_j s_j(\boldsymbol{\omega})\right) \\ A\boldsymbol{\omega} = \dot{\boldsymbol{b}} \\ \boldsymbol{\omega}_j \geq 0 \qquad j \notin \hat{\beta}. \end{gathered} \tag{12}$$

A basic optimal solution to this problem gives the best linearly estimated basis for simulating forward. (Note that there may be a better choice of basis, particularly if the constraint functions $\boldsymbol{b}(t)$ are highly non-linear). The task is then to efficiently solve this (notably non-linear) min-max problem in such a way as to finish with a basic feasible solution.

To do this, we can iteratively reduce the maximum by performing a single pivot on the following LP:

$$\begin{gathered} \min_{\boldsymbol{\omega}} \left(\boldsymbol{e}_{j_{max}} \cdot \boldsymbol{s}(\boldsymbol{\omega})\right) \\ A\boldsymbol{\omega} = \dot{\boldsymbol{b}} \\ \boldsymbol{\omega}_j \geq 0 \qquad j \notin \hat{\beta} \\ s_k(\boldsymbol{\omega}) \leq p_k, \qquad k \neq j_{max} \end{gathered} \tag{13}$$

where j_{max} is the index of the current largest s_j and the p_k are chosen to prevent "whack a mole" in which the pivot increases the minimal maximum. If we choose $p_k = s_{j_{max}}$ at each iteration, we will find a local minimum. However, we may end up with a solution that is not a basic solution for the original problem (eq. (9)). The set of constraints $s_k(\boldsymbol{\omega}) \leq p_k$, $k \neq j_{max}$ will be added as basic and must remain basic so the corresponding slacks can be removed from the solution after pivoting. Notice that this still doesn't entirely prevent "whack a mole", because the maximum may not decrease. We have only insured that it will not increase. In practice, this did not improve the method .

REFERENCES

[1] Jon Lee. A first course in linear optimization, 2016. URL `https://github.com/jon77lee/JLee_LinearOptimizationBook/blob/master/JLee.4.07.zip`.
