# Supplementary material for: Inferring microbial interactions with their environment from genomic and metagenomic data
Source: PLoS Comput Biol. 2023 Nov 13;19(11):e1011661. doi: 10.1371/journal.pcbi.1011661 (PMC10681327; doi:10.1371/journal.pcbi.1011661)
Supplement: Attachment — Submitted filename: response.pdf [file pcbi.1011661.s003.pdf]

Response to Reviewers.

James D. Brunner[1,2], Laverne A. Gallegos-Graves[1], and Marie E. Kroeger[1,3]

[1]**Biosciences Division, Los Alamos National Laboratory, Los Alamos, NM, USA**
[2]**Center for Nonlinear Studies, Los Alamos National Laboratory, Los Alamos, NM, USA**
[3]**Current Affiliation: In-Pipe Technology, Wood Dale, IL, USA**

Author responses in blue

REVIEWER 1

The authors provide a tool – MetConSIN (Metabolically Contextualized Species Interaction Networks) - to efficiently simulate community metabolism in an interpretable way. Computational efficiency is achieved by reducing the number of optimizations performed to calculate the optimal flux in the metabolic network through the transformation of the linear programming problem to a set of differential equations, who's solution can be carried forward in time without the need to re-optimize the parameters of the problem. This computational transformation also paves the way for the second, and main contribution of the new tool, which is interpretability. Because the transformation effectively produces an interaction network between organisms and metabolites, this allows for an immediate visual understanding of the causal interactions leading to metabolite, and ultimately species dynamics in the community.

We would like to thank the reviewer for their thoughtful summary of our work. We agree that interpretability of the model is indeed the main contribution of the tool.

Major comments While I see no technical flaw in this work, I simply do not think it is innovative enough to warrant publication in a journal like PLoS Computational Biology. Many other tools exist that perform similar analysis of genome-scale metabolic models. In fact, excellent tools exists, such as COMETS (which is not cited here for some reason, and I am also not an author of), that are scalable (a major novelty claim in this paper) and even offer spatially resolved simulations. Specifically, COMETS automatically offers similar analysis, where the major difference is the automatic creation of interaction networks in MetConSIN, which is, in my opinion, not enough to warrant publication in a non-specialized journal. The authors also offer no comparison with existing methods (which was done in their previous publication)

While we acknowledge that there exist other tools for investigating microbial communities using GSMs, we believe that the ability to infer a detailed network of interactions directly from the dynamics of DFBA is a novel and useful contribution. We now include a detailed discussion of the new advances that MetConSIN provides in relation to existing methods. In the discussion, we have added the following (lines 418-441):

> To our knowledge, MetConSIN is the only method available for constructing the interaction networks implied by the DFBA mathematical model. It is possible to infer a rough approximation of the networks that MetConSIN provides by simply using the fluxes computed with FBA at a single time-point. However, this has several disadvantages compared to MetConSIN. First, FBA alone cannot accurately describe the effect of each metabolite on the growth rate of each microbe (i.e. arrows from metabolite to microbe). In fact, MetConSIN demonstrates that the concentrations of many metabolites that are consumed by a microbe may be perturbed with no effect on the microbial growth rate. A more accurate picture of the interactions happening within a community at specific point in time (or, equivalently, with a specific metabolic environment) could be produced by repeatedly computing FBA solutions while perturbing metabolites, but this would be extremely computationally expensive. In contrast, MetConSIN immediately provides a picture of which metabolites actually effect the growth of the microbes in a community, i.e. the rate-limiting metabolites. Second, MetConSIN provides information about interactions not just between metabolites and microbes, but also between metabolites and metabolites as mediated by microbes. In other words, MetConSIN shows how one metabolite can be effected by changing the availability of another. Finally, MetConSIN provides details about when, how and why a network changes qualitatively as a microbial community manipulates its environment, whereas a network based directly on flux balance can only provide details for a single discrete time-point. Therefore, discovering the transition points at which a community changes its behavior (i.e. basis changes in MetConSIN) would require relatively dense sampling with FBA.

and (line 480-494)

> As an alternative to networks based directly on the solution to flux balance analysis, it is possible to construct a network of interactions between microbes by simulating knock-out or paired-growth experiments with community FBA approaches like *SteadyCom*[**?**] or *MICOM*[**?**]. However, this approach does not provide details about

> the interactions between microbes and metabolites, and assumes the community to be at equilibrium. Likewise, other DFBA tools, for example *COMETS*[?], can be used to infer some interactions by simulation of organisms in different combinations and with *in-silico* genetic modifications. In this way, *COMETS* was used to infer interactions within small 2 and 3 member communities [?]. However, this approach required repeated simulation to show that all community members needed to be present for growth and thus interactions were taking place, and inference of the interactions themselves was based on foreknowledge that allowed simulated gene knockout experiments. In contrast, MetConSIN, infers interactions from a single simulation without a need for further simulated experimentation to determine the details of the interactions.

or with real world data, which make this paper quite "thin".

In order to provide comparison with real-world data, we identified a publication (Weiss et al., citation 54 in the manuscript) that coupled pair growth experiments with inference of genome-scale metabolic models. We use the models and data provided by that paper to show the accuracy of our method, and discuss reasons for discrepancies between observed growth and our predicted growth and interactions. This analysis appears in a new subsection entitled "Simulation of growth experiments & empirically observed 381 interactions".

A comparison of how the interaction networks differ between their smooth simulations and numerical solvers would go a long way.

In order to compare our interaction networks, which are built from a piece-wise smooth dynamical system, and the interaction networks implied by flux balance analysis, we used FBA to compute interaction networks using the simple heuristic that assumes a microbe's growth is effected by any metabolite that it consumes. Comparison of the resulting networks is detailed in the manuscript (lines 442-464):

> To demonstrate the difference in networks inferred directly from FBA and networks constructed by MetConSIN, we created a method to infer a network from FBA by considering the fluxes of each reaction. These fluxes indicate how a microbe affects a metabolite, and so we added edges from microbe to metabolite weighted by the value of each flux. FBA provides no obvious way to assign edges from metabolites to microbes, so we assigned an edge from a metabolite to a microbe if the microbe consumed the metabolite. This reflects the assumption that a microbe will consume only the metabolites necessary for its growth. We note that such an edge should not be interpreted in the same way as a metabolite to microbe edge inferred by MetConSIN, which indicates that the metabolite in question directly affects the growth rate of the microbe (i.e. is rate limiting). We computed MetConSIN and direct FBA networks for a sample of small (2-4 member) communities chosen randomly from the 12 soil isolates. Figure 7 shows that there is some difference in weight of shared edges between MetConSIN networks and those constructed from FBA, and that networks constructed from FBA often contain many additional edges in comparison to MetConSIN networks.
>
> As a result of the choice to consider every consumption of a metabolite indicated by FBA to give an edge from metabolite to microbe, networks inferred directly from FBA imply competition for many metabolites that are not rate limiting. These competitions in turn imply strong negative interactions between microbes when this is not truely the case. Table 3 shows how MetConSIN identifies a set of species-species interactions based only on rate-limiting metabolites (note that in simulation oxygen was initially scarce), whereas Table 4 incorrectly identifies many additional interactions mediated by a host of metabolites that do not in fact limit growth of any microbe.

as well as figure 9.

Finally, I think the figures with the networks would benefit from simplification. All of the nodes that are only connected by insignificant links (grey) could be removed, where the full network would be found in some supplementary figure. This would make reading the labels much easier and the interpretation and comparison of the different panels much easier, in my opinion.

To make the network figures more readable, we redrew the networks with a transparency filter for the edges and nodes that do not have a meaningful impact on community organization or metabolism (metabolites that do not significantly change value or impact microbial growth).

Minor Comments

- Line 146: Should it be "with non-decreasing $c_{ij}^2(y_j)$"?
- Line 184: non → not
- Line 340: Delete the word "in" in the sentence containing "but the strength of the competition may vary in with different"

These corrections have been made.

REVIEWER 2

The authors developed a mathematical approach that can extract microbe-metabolite interaction network from dynamic flux balance analysis (dFBA). This approach is based on their previous method to convert dFBA into piecewise ODEs. Various networks were constructed by interpreting the structure of ODEs. This is very interesting approach and I am very excited about it. I only have a few minor comments:

We would like to thank the reviewer for their interest and excitement.

1. Each optimization step in dFBA is not unique in terms of which metabolites are consumed and produced. Are the equivalent ODEs unique or not? I am not familiar with the details of SurfinFBA; I guess the uniqueness maybe related to the choice of index set B. Since the reconstructed metabolic networks are derived from these ODEs, are they unique or not? It would be great if the authors add a comprehensive discussion about the uniqueness of dFBA, equivalent ODEs, and the reconstructed networks.

The reviewer is correct that SurfinFBA suffers from similar uniqueness problems as DFBA. We have added the following discussion (lines 556-576):

> In addition to limitations of the underlying GSMs, interpretation of MetConSIN's results must also account for the inherent limitations of flux balance analysis. Whenever FBA is computed, it is possible that the optimal set of fluxes found is not unique. Like many methods based on FBA, MetConSIN attempts to mitigate this problem with a secondary optimization, by default minimizing the total flux through the metabolism of each taxa. Unlike other dynamic FBA methods, which must choose between non-unique solutions at every time-step, MetConSIN must only choose between optima at the very initial point of the simulation. However, MetConSIN will encounter non-uniqueness of the choice of basis for forward simulation at its discrete optimization (basis-change) time-points, meaning the resulting ODEs and networks are also non-unique. When this happens, the optimal solution found by MetConSIN is known as "degenerate", and it is exactly when the solution is degenerate that MetConSIN may change basis — without this degeneracy there would be no alternative basis to switch to. If only one possible basis allows forward simulation, MetConSIN will choose that basis and resulting networks. However, if the choice of basis is still non-unique even when constrained to allow forward simulation, MetConSIN chooses a basis by attempting to maximize the time until another basis is needed (details of this maximization can be found in supporting file `S3_Details.pdf`). This maximization is based on local (in time) information available to MetConSIN, reflecting the idea that a microbe will choose a strategy that will be more likely to work for a longer time based on the systems current state.

2. The rationale of developing this approach needs to be beher explained. The optimal solution at each time point in dFBA depicts a community-level metabolic network: the exchanged metabolites of each GSM are known and their rates are quantitatively solved. Then why did the authors develop an indirect method to infer the network which requires conversion of dFBA to ODEs? I understand that ODEs are way easier to solve than dFBA but, in terms of network construction, how do you compare the pros and cons of the two approaches? And how different are between the metabolic networks inferred between the two approaches?

While the effect of each microbe on its environment (i.e. microbe $\rightarrow$ metabolite edges in the network) are known from FBA, the networks provided by MetConSIN provide a few key advantages. First, FBA cannot provide an exact picture of the effect the environment has on the growth rate of the microbe (i.e. the metabolite $\rightarrow$ microbe edges). Second MetConSIN reveals a more fundamental interaction that includes the effect of metabolites on each other. Third, MetConSIN reveals when fundamental changes in interactions occur. Please see our response to reviewer 1 for the significant additions we have made in the manuscript detailing the difference between networks constructed from FBA and those constructed by MetConSIN.

3. Both dFBA and ODEs are first-principle approaches. It would be super interesting if the approach can be extended to introduce constraints from metabolomics data. Could you discuss the possibility of integrating dFBA/ODEs with metabolomics data to infer realistic metabolic networks?

We agree with the reviewer that MetConSIN could benefit from metabolomic data in order to constrain the environment ($\boldsymbol{y}$ in the model) which ultimately determines the interactions inferred. We now include the discussion (lines 529-539):

> In the *in silico* experiments we performed with our 10 soil isolates, we lacked detailed information about an environment on which the interactions would take place. We assumed that every metabolite that the models could make use of was available, and calculated interactions based on this "uniform" media. For more precise study of known systems, the model can be constrained by information about the available environmental metabolites, e.g. using metabolomics data. We demonstrate this with our experiments using the data from Weiss et al.[**?**], in which the authors used metabolomic data to define a model environment. Alternatively, when modeling a well studied environment, environmental information can at times be found in the literature. In particular, the *Virtual Metabolic*

Human project [?] define a number of environments corresponding to common human diets (e.g. "E.U. Average" or "High Fiber").

REVIEWER 3

In this paper, the authors develop a nice method to perform dynamic FBA on multispecies microbial communities that is efficient and offers a time varying view of the interactions underlying the species and metabolites. Current dynamic FBA methods are time consuming and typically fail to offer insights into the interactions governing the dynamics. The present method overcomes both these limitations. The conceptual advance is in recognizing that the optimization problem governing FBA can be mapped to a system of linear equations and hence translated to a system of ODEs with parameters that remain constant over finite subintervals of time. The parameters change when metabolite concentrations change enough to violate the constraints on the original FBA problem. The authors present an elegant description of this new formalism and a software, MetConSIN, for implementing it. They apply it to a set of 10 soil microbial species, whose genomes they identify by sequencing and then construct genome scale metabolic models of each of the species to be used in MetConSIN. They deduce the interactions governing the species and the regimes over which the networks change. The proposed method, in my opinion, represents a significant advance over existing methods because of its ability to offer time varying interaction maps and hence insights not readily gained by existing methods. The computational gains are a bonus.

We would like to thank the reviewer for their thoughtful reading of our manuscript and accurate summary.

The paper is well written overall. I have a few comments for the authors to consider.

Major comments:

1. My first comment is with respect to benchmarking. While the authors demonstrate the applicability of their method to the 10-member soil community, they do not show how their method compares with existing methods, particularly the dynamic FBA set up in Eqs. (1)-(4). Are the time courses predicted in Fig. 2 similar to what might be expected from Eqs. (1)-(4)? If the 10-member community is cumbersome to solve, can a smaller subcommunity – even a 2 member community – be studied to establish the similarities and differences between the present method and the existing formalisms?

We agree that this comparison and a discussion of it are a valuable addition to the manuscript. We now include a lengthy discussion of the theortical differences between approaches. Furthermore, we directly compare the approaches by simulating with a "direct" method based on Eqs. (1)-(4). Please see our response to reviewer one for our added discussion of the differences between the networks produced. Additionally, we compared the time-course simulations themselves to verify that our method is faster while provided similar solutions. We have added the following to the manuscript (lines 465-479):

> In Brunner & Chia [?], we demonstrated the theoretical improvement offered by *SurfinFBA*, the DFBA algorithm at the core of MetConSIN, by showing that *SurfinFBA* requires far fewer linear optimizations than a direct method of solving DFBA with a standard ODE solver, as well as a similar method that also makes use of forward simulation bases [?]. We used this metric of performance because it allows us to compare the theoretical algorithms behind available solvers, regardless of improvements gained by choice of language or code optimization.
>
> In order to benchmark the time improvements provided by *SurfinFBA* and demonstrate that the solutions provided are similar to a direct repeated optimization approach, we implemented a direct solve method using *scipy*'s `solve_ivp` function[?]. This is fundamentally the same as the method suggested in the documentation of the CobraPy Toolbox [?]. We simulated the DFBA problem using MetConSIN and the direct method for a sample of small (2-4 member) communities chosen randomly from the 12 soil isolates. In figure 8, we show that *SurfinFBA* is indeed faster than the direct method and that solutions are very similar, with some slight error.

as well as figure 10.

2. Along the same lines, is a comparison with any experimental system feasible? The authors seem to have cultured the 10-members they studied. Could their growth rates be monitored in multi-species cultures and then compared with corresponding model predictions? If co-culturing is not possible, are other previously published datasets amenable to comparisons with the present model? If this not possible too, the authors must discuss this and mention explicitly what prevents comparisons with experiments. The difficulty may exist with current methods too, in which case, this may not be a limitation of the present study alone, but it must be discussed nonetheless.

Unfortunately, our own experimental work with the 10 soil isolates is still ongoing. However, at the reviewers suggestion we have identified a previously publish set of experiments in which the authors grew 12 taxa in pairs. Fortuitously, the authors of that work performed metabolomics and genomics and constructed genome scale models for their strains. We now include simulation of that community as a whole as well as the monocultures and pairs that were included in the original work. Our results demonstrate the limitations of DFBA as a predictive model, but also the strength of MetConSIN to interpret

the mechanisms of microbial interaction. A full discussion can be found in the newly added section of our manuscript titled "Simulation of growth experiments & empirically observed interactions".

3. The analysis of the interaction networks and their evolution (Figs 2-4) is very nice. It highlights the strength of the method. I felt though that the interpretation of the transitions seen seemed somewhat superficial. The authors mention that the first transition is when bc1012 altered its connectivity three times in quick succession (lines 286- 288). They, however, do not provide any explanation of these changes in connectivity. Thus, while knowledge of these transitions is indeed an advance over existing models and is thus welcome, a mechanistic understanding of the transitions based on the metabolic models of the species and the nutrients available could have been more satisfying. Are these explanations forthcoming? If not, the authors must discuss why.

We would like to thank the reviewer for this comment because it suggested to us a feature which we could easily add to MetConSIN but had previously overlooked. MetConSIN now provides three pieces of information which shed light on the nature of the transitions in the model. Specifically, we now return (1) a dictionary detailing exactly which internal reaction bounds were violated causing a transition, (2) a dictionary detailing the differences (before/after) between the bases used for forward simulation (which can be inspected to discover active reactions/constraints) and (3) a table containing the differences (before/after) in the network itself. We demonstrate these new features in the manuscript as follows (lines 3116-333):

> The two major transitions in the simulation both involved a series of basis-changes, meaning that one or more microbes altered their connectivity in the network, and MetConSIN can provide details as to why and how these transitions happened. For example, in the first transition, in which the model of genome *bc1012* altered its connectivity, MetConSIN reports that the first transition happened because the solution violated the upper bound of glucose uptake. Prior to this transition, glucose was abundant enough that it did not limit *bc1012* growth. After this transition, the constraint on glucose uptake was activated by the model along with the reaction for lactate oxidation. The second transition occurred immediately after the first, when the reaction dehydrogenating NADH violated its lower bound, causing deactivation of this reaction and the fumarate exchange upper bound constraint. The result of these internal changes can be observed in the interaction networks simply by inspecting the difference in the network edge weights (where we may assign the weight 0 to an edge that is not present in a network). The largest (in magnitude) changes were in *bc1012*'s production of succinate and consumption of fumarate, both of which were reduced by about 77%. We display which microbe changed its network connectivity at each transition with the style and color of the dashed lines in fig. 2 that indicate the time-points at which the transitions occurred.

4. My final comment is on the way interactions between species are deduced (Eq. 13). Pairs of species are chosen and their interactions mediated by metabolites are summed with suitable weights to yield the net interactions between the species. This method yields pairwise interactions. However, species often experience high-order interactions (e.g., see: 1) https://www.pnas.org/doi/10.1073/pnas.1809349115; 2) https://www.nature.com/articles/s43588-021- 00131-x). Does the present method thus miss these high-order interactions? Because the dynamic FBA formalism does not make any assumptions on the interactions but only deduces them, any high-order interactions present must exist in the model calculations. The deduction method may have to be changed to consider triplets of species, quadruplets of species, etc. (instead of just pairs) in order to deduce third-order, fourth-order, etc. interactions. I wonder if currently, the pairwise interactions deduced yield 'effective' pairwise interactions, as has been suggested in the recent study above (https://www.nature.com/articles/s43588-021-00131-x)? Again, I feel that the authors must at least comment on high-order interactions, given their possible presence in multi-species communities and the focus of the present study on deducing interaction networks.

We agree with the reviewer on the two important points that their comment raises. The first is that our specie-species networks only provide a rough approximation to the full set of interactions implied by the species-metabolite network. The second is that higher order interactions are indeed an important consideration. We now include the following discussion in the manuscript (lines 540-555):

> Higher-order interactions within microbial communities likely play an important role in community organization [**? ?**]. However, the species-species interaction networks that we provide are fundamentally pairwise, and lack the capacity to convey the higher-order interactions that may be implied by the species-metabolite networks they are built from. In fact, any species-species model will lack the complexity necessary to reflect the possible interactions of a species-metabolite model [**? ?**]. However, MetConSIN includes not just a single network, but a series of them. This means that it has the capacity to capture higher-order interactions that emerge from the transitions between network structure. These represent changes in pairwise interactions caused by the actions of the community. Inferring higher order interactions or "effective" pairwise interactions that include higher-order effects[**?**] directly from the series of MetConSIN species-metabolite networks remains an ongoing area of research. We note also that higher-order effects are often mediated by secondary metabolites [**?**], which may be missing from the GSMs input

> into MetConSIN. Improving GSMs and automatic generation of GSMs remains an active area of research, and MetConSIN will benefit immediately from any improvements made in that area.

Minor comment:

1. On lines 126-131, the authors indicate that the method to choose the matrices (Bik) are outlined elsewhere. For completeness, I feel that the authors may wish to provide a brief outline of how this choice is made.

As the details are very technical, we wished to keep our discussion of this process as brief as possible. To that end, we added the following to the manuscript (lines):

> In brief, it is necessary to solve a new optimization problem defined by the time-derivative of the constraints of the original FBA linear program whenever a new B_i^k is needed.

We do provide full details in the supplemental materials.

2. On lines 140-142, the authors mention that holding the metabolite levels constant would yield a snapshot of the interaction may between the species. Could this be shown? Also, I would imagine that the species compositions would evolve with time even if the metabolite levels were held constant. Then, would the interaction map not also change? The authors may wish to comment on this.

We apologize for the confusing way this line was worded. We have replaced it with the following (lines 142-148):

> Furthermore, the FBA solutions for each community member, and as a result the interactions that can be inferred, depend entirely on the metabolic environment ($\boldsymbol{y}$). Therefore, for a fixed metabolic environment (created by, e.g., flowing metabolites into a bioreactor at an increasing rate or finding a chemostatic community equilibrium) DFBA provides an interaction network representing community metabolic activity.
